# Subwavelength-scale nanorods implemented hexagonal pyramids structure as efficient light-extraction in Light-emitting diodes

**DOI:** 10.1038/s41598-020-62257-8

**Published:** 2020-03-26

**Authors:** Jae Yong Park, Buem Joon Kim, Chul Jong Yoo, Wan Jae Dong, Illhwan Lee, Sungjoo Kim, Jong-Lam Lee

**Affiliations:** 1 Department of Materials Science and Engineering, Pohang University of Science and Technology (POSTECH), Pohang, 790-784 Korea; 2 Division of Advanced Materials Science, Pohang University of Science and Technology (POSTECH), Pohang, 790-784 Korea

**Keywords:** Lasers, LEDs and light sources, Materials for optics, Nanoscale materials

## Abstract

Subwavelength-scale nanorods were implemented on the hexagonal pyramid of photochemically etched light-emitting diodes (LEDs) to improve light extraction efficiency (LEE). Sequential processes of Ag deposition and inductively coupled plasma etching successfully produce nanorods on both locally unetched flat surface and sidewall of hexagonal pyramids. The subwavelength-scale structures on flat surface offer gradually changed refractive index, and the structures on side wall of hexagonal pyramid reduce backward reflection, thereby enhancing further enhancement of the light extraction efficiency. Consequently, the nanorods implemented LED shows a remarkable enhancement in the light output power by 14% compared with that of the photochemically etched LEDs which is known to exhibit the highest light output power. Theoretical calculations using a rigorous coupled wave analysis method reveal that the subwavelength-scale nanorods are very effective in the elimination of TIR as well as backward reflections, thereby further enhancing LEE of the LEDs.

## Introduction

Light-emitting diodes (LEDs) have rapidly replaced conventional light sources because of their high efficiency, long lifetime, and environmental friendliness^1–3^. In particular, group III-nitride-based vertical LEDs (VLEDs) are promising candidates for high power applications including general illumination^4,5^. Although internal quantum efficiency (IQE) of the LEDs has been achieved to nearly 80%^6,7^, much room remains for enhancement of the light extraction efficiency (LEE), so that they can serve as next-generation light sources. Limited LEE originates from the total internal reflection (TIR) at the interface of GaN with air, resulting in confinement of photons inside the active layer of LEDs^8–10^.

To eliminate TIR and extract confined photons inside the active layer of the LEDs, numerous methods of surface roughening and texturing have been explored such as growth of nanorods^8,10–12^, dry-etching^9,13^, and wet-etching process^5,14^. The ZnO nanorods grown by hydrothermal method were widely used to increase LEE. However, additional seed layer^15^ or surface treatment^10^ were required to produce highly dense nanorods. Moreover, the planar ZnO seed layer could induce TIR at the interface of GaN (*n* = 2.5) with ZnO (*n* = 2.0), so it could not efficiently reduce the TIR. To eliminate the TIR between two different materials, the dry etching methods have been developed because they could directly implement homogeneous GaN nanostructures on the GaN surfaces. However, forming and removing of dry etching mask were indispensable to produce nanostructure, resulting in complex process and low throughput.

Among the methods that solve these problems, surface roughening of the nitrogen (N)-face GaN (000–1) of V-LEDs using photochemical etching (PCE) is known to be the most efficient way due to simple process of wet etching and the largest enhancement of the LEE up to 2–3 times compared to the other methods (Table S1). Thus, the PCE have been used as the standard process in the solid-state lighting industry. The PCE allowed to produce wavelength scale hexagonal pyramid structures with sidewall angle of 31.6° on the surface of GaN without surface treatment due to the selective etching of the GaN {10-1-1} surface which has the lowest surface energy^16,17^. The hexagonal pyramid structure exhibited the highest enhancement (>300%) in light output power compared to flat GaN surfaces. However, there was a flat region on the surface locally where is unetched during the PCE process. This could lead to induce TIR. Moreover, the backward reflections at the side wall of hexagonal pyramid also contribute to decrease LEE (Fig. S1). The sidewall angle of 31.6° was known not to be an optimal sidewall angle for the light extraction^9^ because 50% of incident light was reflected at the side wall of hexagonal pyramid (Fig. S1b,c). Such problems can be solved by employing subwavelength-scale nanorods on both the flat surface region and the side walls of hexagonal pyramid. The formation of subwavelength-scale structures on the flat surface could offer a gradually reducing refractive index to air, and the subwavelength-scale structures on side walls of hexagonal pyramid could decrease he backward reflection, thereby enhancing further enhancement of the LEE.

In here, we demonstrated the fabrication of subwavelength-scale nanorods on the hexagonal pyramids structure to maximize the LEE. Thin Ag film was deposited on the photochemically etched GaN, followed by inductively coupled plasma (ICP) etching with chlorine (Cl_2_). The Ag reacted with Cl radicals, producing AgCl nanodots during ICP etching. Due to the spontaneously produced AgCl nanodots acting as an etching mask, subwavelength-scale nanorods were successfully produced on both a flat surface region and the side walls of hexagonal pyramids. Consequently, LED with the nanorods implemented hexagonal pyramids structure shows a remarkable enhancement in the light output power by 14% compared to that of the photochemically etched LEDs which is known to exhibit the highest light output power. Theoretical calculations using a rigorous coupled wave analysis method reveal that the subwavelength-scale nanorods are effective in the elimination of total internal reflection (TIR) as well as backward reflections, thereby further enhancing LEE of the LEDs.

## Results

### Fabrication of subwavelength-scale nanorods implemented hexagonal pyramid

**Figure 1:**
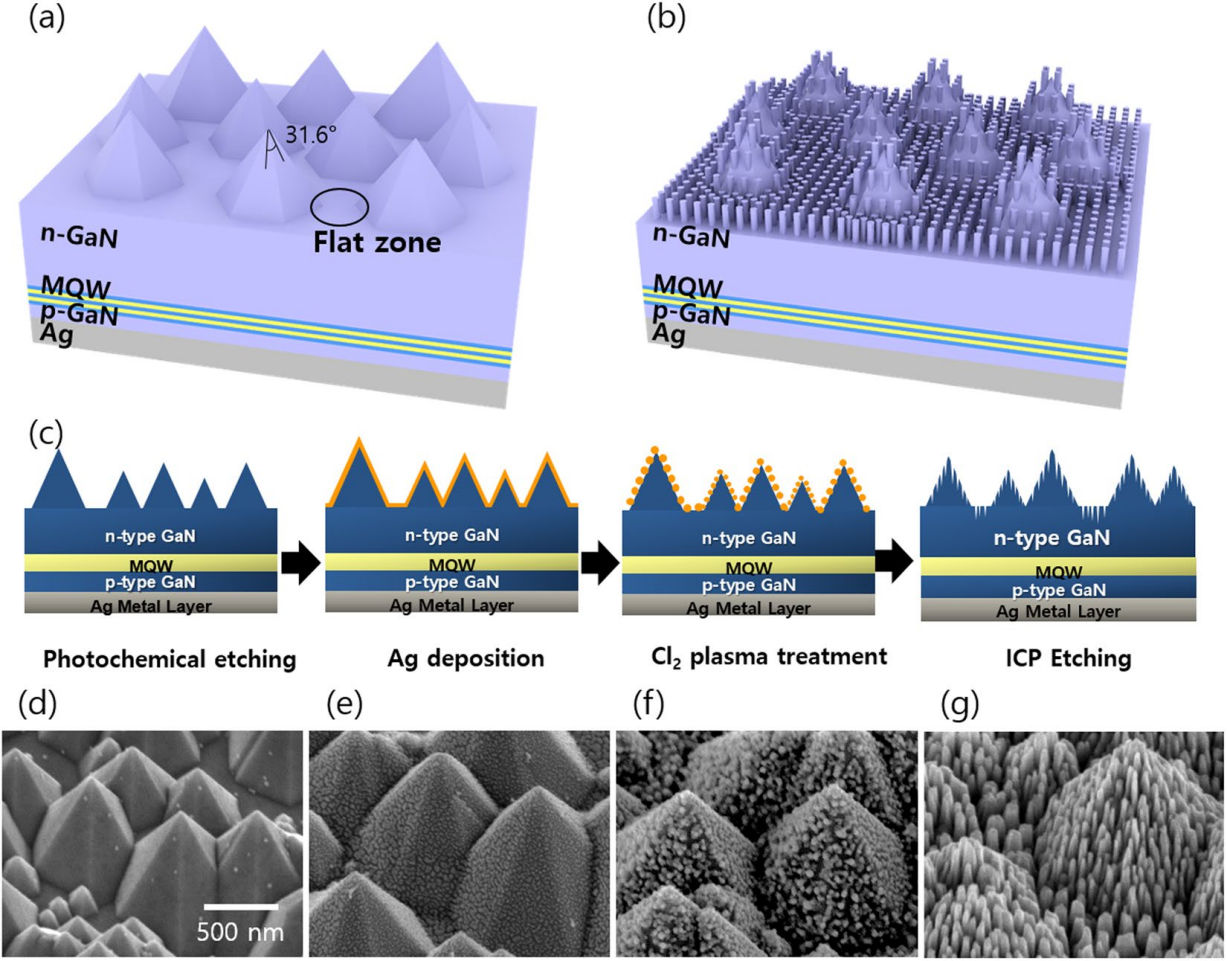
Schematic illustration of (**a**) photochemically etched LEDs and (**b**) subwavelength-scale nanorods implemented LEDs. (**c**) Schematic fabrication process of implementing nanorods on both flat side and sidewall of hexagonal pyramid. Scanning electron microscopy (SEM) images of the surfaces of n-GaN after (**d**) photochemical etching, (**e**) deposition of Ag, (**f**) Cl_2_ plasma treatment, and (**g**) ICP etching.

Figure 1a is a schematic illustration of the photochemically etched GaN-based LED devices. Although hexagonal pyramids structures were produced through the PCE process, there was a flat region of 10% producing inevitably due to a non-uniform wet chemical etching (Fig. S3). The flat zone could induce TIR, resulting in insufficient enhancement of LEE. In contrast, the subwavelength-scale nanorods implemented on the pyramids structures (Fig. 1b) could effectively enhance LEE by reducing TIR at the flat zone as well as by eliminating the backward reflection at the side wall of the hexagonal pyramids. This is due to that the nanorods provide a gradual decrease of refractive index from GaN to air. Figure 1c showed the schematic drawing of fabrication steps to implement the subwavelength-scale nanorods (see the Experimental Section for detailed sample preparation). First, a planar VLED was photochemically etched in KOH solution to produce hexagonal pyramid structure on surface of the GaN (Fig. 1d). The selective etching of the GaN {10-1-1} surface allowed to form the hexagonal pyramid structures on the surface of the GaN because the surface has the lowest surface energy and the smallest number of bonds at the surface^18^. Then, 5-nm-thick Ag was deposited on the photochemically etched GaN by using e-beam evaporator (Fig. 1e). The high surface energy of Ag makes the Ag nanodots to be discontinuous. Next, ICP etching was performed to produce subwavelength-scale structures. At the initial stage of the ICP etching (Fig. 1f), Ag nanodots reacted with Cl radicals to produce AgCl nanodots^19^. These spontaneously produced AgCl nanodots acted as an etching mask during ICP etching. After the ICP etching, residual AgCl compounds were easily removed by hydrochloric acid. Then, the subwavelength-scale nanorods were implemented on the hexagonal pyramid structures (Fig. 1g).

### Size tunability of subwavelength-scale nanorods

**Figure 2:**
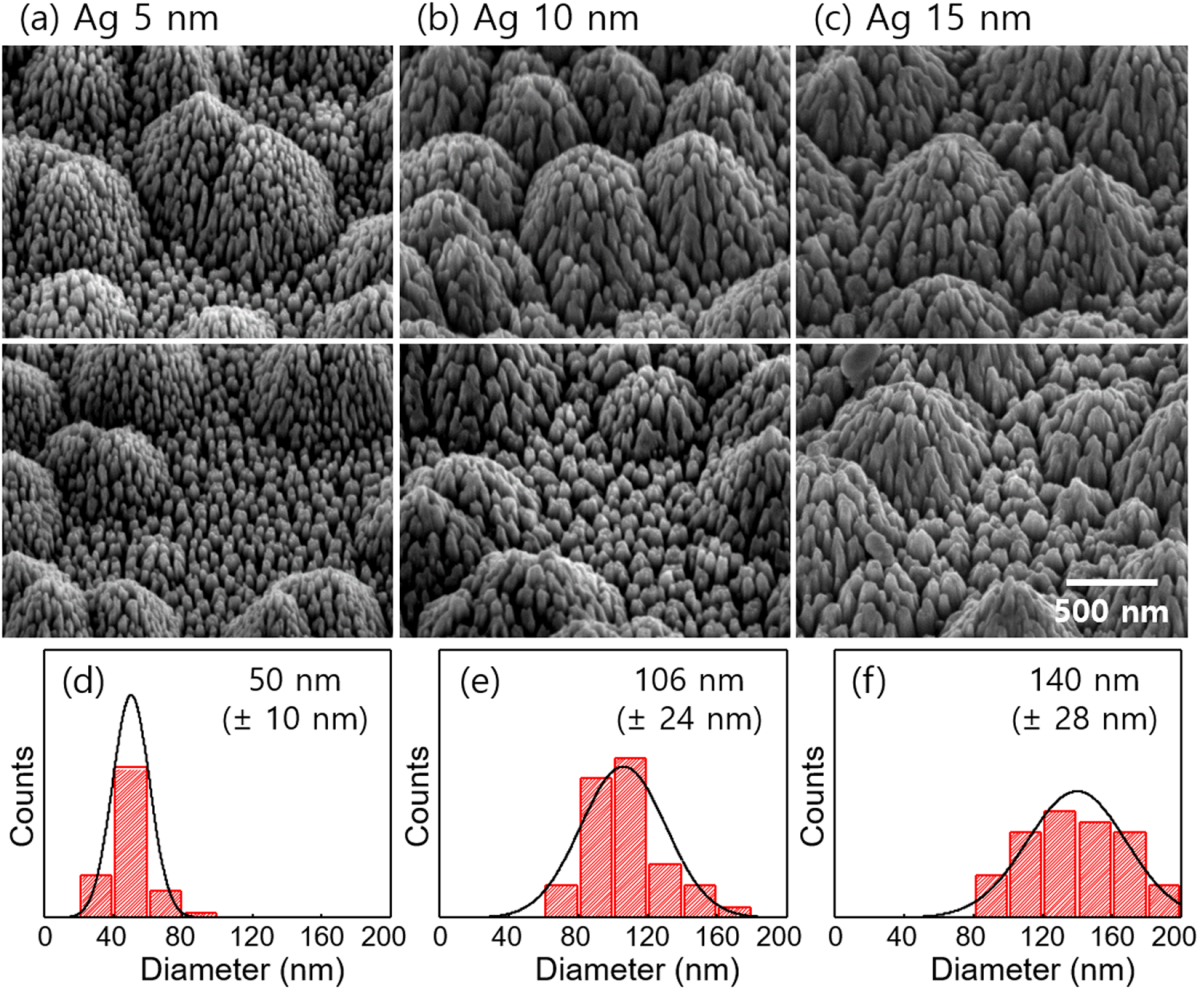
Scanning electron microscopy (SEM) images of subwavelength-scale nanorods with (**a**) 5-nm-thick Ag, (**b**) 10-nm-thick Ag, and (**c**) 15-nm-thick Ag. (Top) enlarged view of hexagonal structure and (bottom) flat surface. The histogram of diameter of nanorods with (**d**) 5-nm-thick Ag, (**e**) 10-nm-thick Ag, and (**f**) 15-nm-thick Ag.

The vertically aligned nanorods were produced on both flat side and side wall of hexagonal pyramid due to an anisotropic ICP etching of GaN (Fig. 2a–c). The diameter of the subwavelength-scale nanorods (*d*_NR_) can be controlled by changing the Ag thickness. When 5-nm-thick Ag was used, the average *d*_NR_ was 50 nm (Fig. 2d). As the Ag thickness is thicker, the *d*_NR_ increased up to 140 nm (Fig. 2e,f). Note that the standard deviation of *d*_NR_ was also increased from 10 nm to 30 nm as the Ag thickness thicker, which is attributed to the aggregation of the nanorods.

### Optical properties of subwavelength-scale nanorods

**Figure 3:**
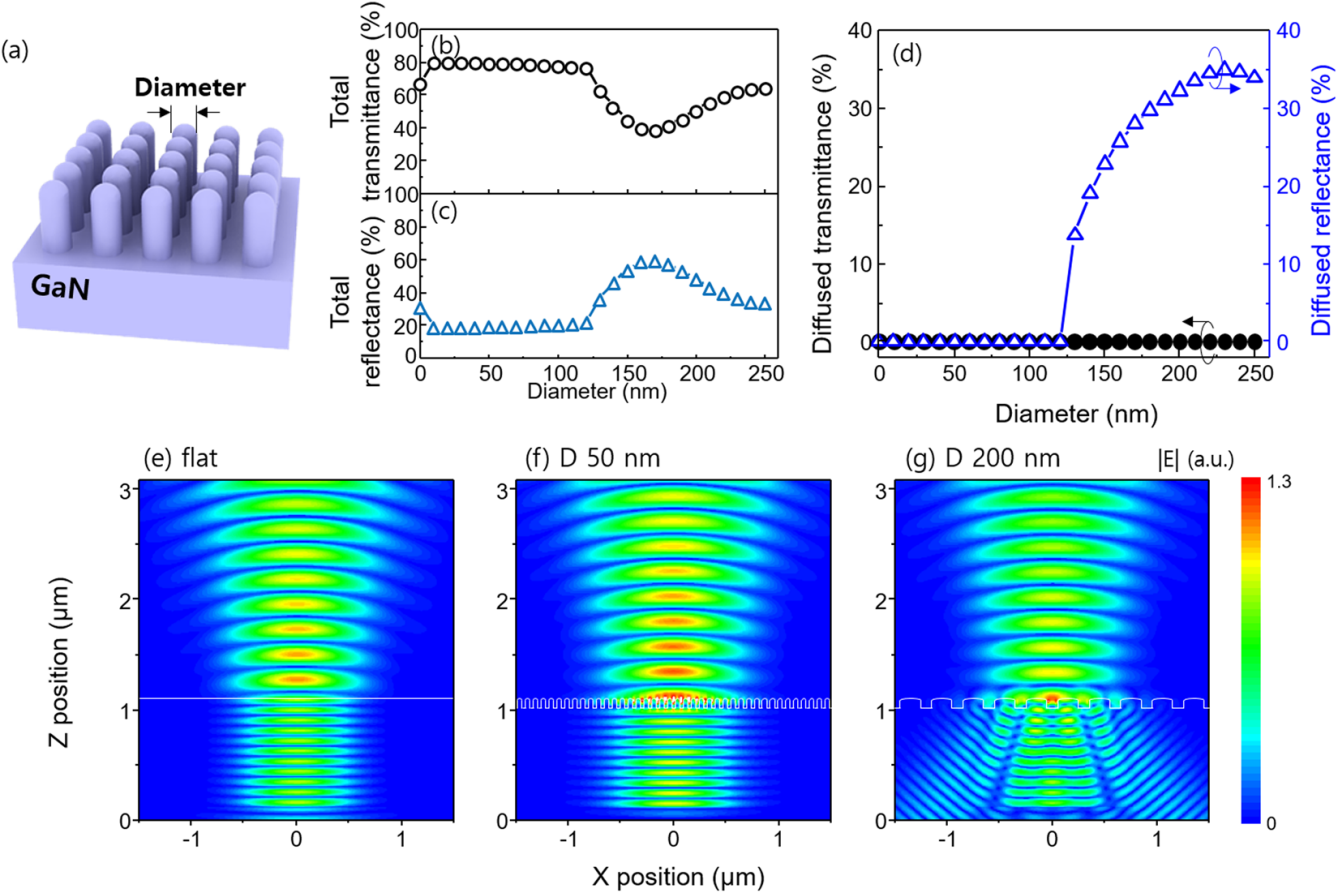
(**a**) Schematic illustration of calculated structures. Calculated (**b**) total transmittance and (**c**) total reflectance spectrums of nanorods implemented GaN at a wavelength of 450 nm. (**d**) Calculated diffused transmittance and diffused reflectance of nanorods implemented GaN as a function of diameter of nanorods at a wavelength of 450 nm. Cross-sectional electric field distribution of (**e**) flat GaN, nanorods implemented GaN with diameter of (**f**) 50 nm and (**g**) 200 nm.

The *d*_NR_ played a critical role in optical properties of GaN. Rigorous coupled wave analysis (RCWA) was used to investigate the effect of *d*_NR_ on the transmittance of the GaN. Figure 3a showed schematic illustration of GaN with subwavelength-scale nanorod. The height of the nanorods was set to be 100 nm, and the gap of the nanorods was set to be half of the *d*_NR_.

We calculated total transmittance (*T*_total_, Fig. 3b) and total reflectance (*R*_total_, Fig. 3c) at a wavelength of 450 nm. The *d*_NR_ = 0 represented flat GaN without nanorods. The flat GaN exhibited *T*_total_ = 68%. When *d*_NR_ < 120 nm, *T*_total_ was calculated to be higher than 77%, which is higher transmittance compared to planar GaN surface because nanorods provide smooth refractive index transition from GaN to air^19,20^. Meanwhile, the *T*_total_ decrease when *d*_NR_ > 120 nm. The decreased *T*_total_ was mainly due to increased *R*_total_ (Fig. 3c). The increased *R*_total_ can be explained by reflective scattering of the incident light (Fig. 3d). When *d*_NR_ > 120 nm, the diffused reflectance was increased to 34%, whereas diffused transmittance becomes zero. The reflective scattering of the light reduced the *T*_total_.

In order to find the origin of the transmittance change with the diameter of nanorod, RCWA calculation was performed. The cross-sectional electric field distribution showed further evidence of the decreased *T*_total_. In both flat surface (Fig. 3e) and GaN nanorods with *d*_NR_ = 50 nm (Fig. 3f), the light propagated the structures straightly without scattering. The electric field intensity in the air of the nanorods implemented GaN was higher than that of flat GaN, resulting in the drastic increase of total transmittance from 68% to 80% (Fig. 3b) because the nanorods provided gradually changed refractive index from GaN to air. As *d*_NR_ increases to 200 nm (Fig. 3g), no scattering was found in the transmitted light, but only the reflected light is scattered. This led to the decrease of *T*_total_ (Fig. 3b) and the dramatic increase of diffused reflectance (Fig. 3d).

The reflective scattering of light can be theoretically explained by reflective grating equation as given by^19^: sin(*θ*_m_) = *m*λ*n*_GaN_^−^*Λ*^−1^, where *m* is the order of diffraction, *θ*_m_ is the *m*-th diffraction angle, λ is the wavelength of light, *n*_GaN_ is the refractive index of the GaN, and *Λ* is the period of the nanostructures. If *Λ* < λ*n*_GaN_^−1^, all diffraction orders were suppressed except the zeroth-order (*m* = 0), so reflected light was not scattered. However, when *Λ* > λ*n*_GaN_^−1^, the reflected light was scattered. In the case of the InGaN blue LED, *Λ* = 180 nm was criteria of the reflective scattering because λ is 450 nm and *n*_GaN_ is 2.5. Since the gap of the nanorods was set to be half of the diameter of the nanorods, the criteria of the scattering is *d*_NR_ = 120 nm. On the other hand, transmissive diffraction mode was be described by: sin(*θ*_m_) = *m*λ*Λ*^−1^, so criteria of the transmissive scattering is *d*_NR_ = 300 nm. Thus, when *d*_NR_ > 120 nm, light was scattered in the reflective direction, whereas light was not scattered in the transmissive direction, resulting in decrease of *T*_total_. This result indicated that the smaller *d*_NR_ can effectively reduce the Fresnel reflection. Thus, we determined optimized thickness of Ag is 5 nm because 5-nm-thick Ag produced nanorods with diameter of 50 nm.

### Characteristics of nanorods implemented light-emitting diodes

**Figure 4:**
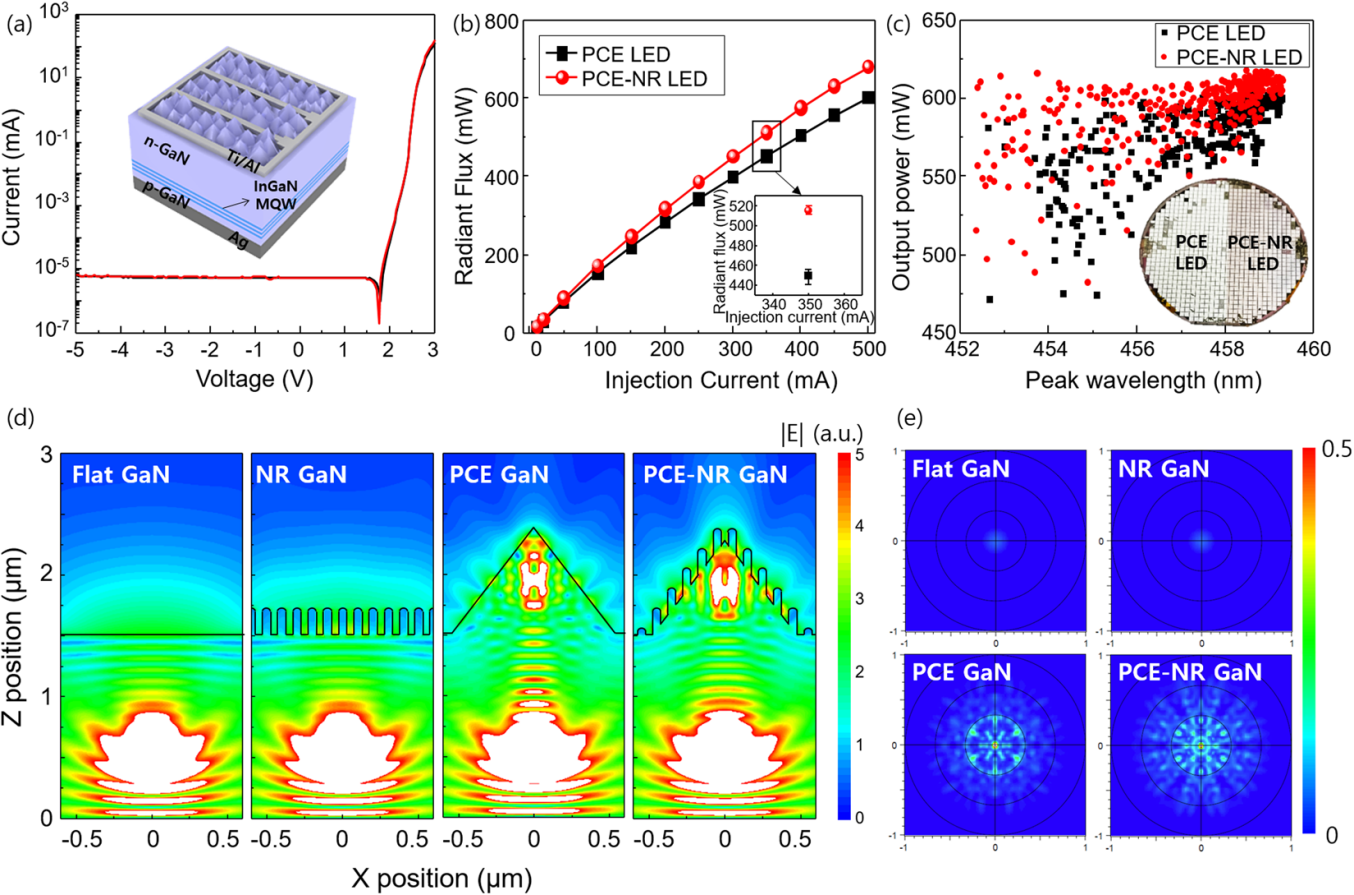
(**a**) Current-voltage characteristics of LEDs and (**b**) radiant flux as a function of injection current. (**c**) Measured light output power of 2-inch wafer, in which nanorods were implemented in only right-half side. (**d**) Cross-sectional electric field distributions and (**e**) polar projection of far-field intensity with different structures; flat GaN, nanorods implemented GaN (NR-GaN), hexagonal pyramid GaN (PCE GaN), and nanorods implemented hexagonal pyramid GaN (PCE-NR GaN).

To investigate the effect of the nanorods on the light extraction of the LEDs with a chip size of 1 ×1 mm^2^, the nanorods implemented on the hexagonal pyramid structure was produced on the top of the LEDs. The 400-nm-thick SiO_2_ was deposited on the GaN to describe encapsulation of the LEDs and protect nanostructures. Figure 4a showed current-voltage (*I*-*V*) characteristics of the hexagonal pyramid LEDs (PCE LEDs) and the hexagonal pyramid LEDs with nanorods (PCE-NR LEDs). The reverse leakage current density at −3 V was measured to be in the range of 5.6 to 5.8 nA mm^−2^, and the forward voltage at 20 mA mm^−2^ was 2.7 V. No distinct changes in electrical properties were found in both LEDs, but the radiant flux of the LEDs increased overall injection current in PCE-NR LEDs (Fig. 4b). For reliable values, radiant flux was measured over 8 LED chips for each samples. The PCE LEDs exhibit radiant flux of 451 ± 7.6 mW, but it remarkably increased to 516 ± 4.2 mW in the PCE-NR LEDs at injection current of 350 mA. The electroluminescence spectrum of the PCE-NR LEDs was also enhanced overall wavelength compared to PCE LEDs (Fig. S2).This process was implemented to 2-inch wafer (Fig. 4c). PCE LEDs were fabricated on the left-half side of 2-inch wafer and PCE-NR LEDs were on the right-half side. The light output power was measured for over 350 LED chips. The PCE-NR LEDs showed enhanced light output power compared to PCE LEDs, indicating that subwavelength-scale nanorods

## Discussion

To investigate the effect of the nanorods implementation on the optical properties for the LEDs, cross-sectional electric field distributions were calculated with flat GaN, nanorods implemented flat GaN (NR GaN), hexagonal pyramid GaN (PCE GaN), and nanorods implemented hexagonal pyramid GaN (PCE-NR GaN) by using finite-difference time-domain (FDTD) simulation (Fig. 4d). The width of the PCE structure was assumed to be 1.2 µm with an unetched flat region of 10% because of locally unetched flat surface (Figs. S3 and S4). The FDTD simulations were carried out under dipole source excitation instead of plane wave excitation to describe unpolarized point source in the LEDs. The calculations were performed under unpolarized light with an equal contribution from two horizontal dipole sources (x, y) and one vertical dipole source (z). The electric field in the air was defined by integrating the electric field from X = −0.6 µm to X = 0.6 µm at the position of Z_max_ (Z = 3 µm). The NR GaN exhibits high intensity of electric field of 86.3 in the air compared to the flat GaN (81.6). The increased extraction of light in the NR GaN was mainly due to gradual refractive index change from GaN to air. In the PCE GaN, the electric field in the air was measured to be 70.5. The low electric field intensity compared to flat GaN was attributed to backward reflection at the sidewall of hexagonal pyramid. In the PCE-NR GaN, the intensity of electric field was remarkably increased to 97.5 because NRs on hexagonal pyramids not only provided gradually changed refractive index from GaN to air but also reduced backward reflection at the sidewall of the hexagonal pyramid.

To calculate far-field radiation patterns and LEEs of the LEDs, the FDTD simulations were carried out in the arrayed structures under dipole source excitation. The LEE was obtained from the ratio of the amount of light output power in the air and the amount of the generated power in the dipole source. For the flat LED and NR GaN, the far-field radiation pattern showed perfect circular symmetry because of TIR at the interface of the GaN with air (Fig. 4e). The light escape angle is very small, and only a small portion of the surface emitted light. The calculated LEEs of the flat GaN and NR GaN were 2.61% and 2.83%, respectively. In the PCE GaN, irregular and scattered radiation pattern was observed because hexagonal pyramid structures extracted confined waveguided light in the GaN. Consequently, the LEE of the PCE GaN was increased to 13.1%. The PCE-NR GaN had higher far-field intensity distribution map spread over the entire surface compared to the PCE GaN, resulting in the high LEE of 15.9%. These results showed that PCE-NR GaN effectively reduced backward reflection at the sidewall of hexagonal pyramid.

In summary, this paper reports a novel method of improving LEE by simultaneous implementing of subwavelength-scale nanorods on both the side wall of hexagonal pyramid and flat surface region, which offers gradually changed refractive index and reduce backward reflection. The three-dimensional structures result in increase of light output power of 516 mW at an injection current of 350 mA; this result is 14% higher than the PCE method. This subwavelength-scale structure implementation method could be used in other optical devices such as organic LEDs, and inorganic solar cells because of simple and easy fabrication with large-area capability.

## Methods

### Fabrication of VLEDs

500-nm-thick undoped GaN buffer layer, a 4-µm-thick n-type GaN, an InGaN/GaN MQW active region, a p-type AlGaN electron blocking layer, and a p-type GaN layer) was grown by metal–organic chemical vapor deposition on c-plane sapphire substrates. The sample was treated with piranha solution (H_2_SO_4_:H_2_O_2_ = 1:1) and boiling aqua regia (HCl: HNO_3_ = 3:1) for 10 min to remove the surface oxides^21^. The Ag-based reflective p-type ohmic contact was then deposited on the p-type GaN, followed by annealing at 400 °C for 2 min in ambient air. Diffusion barrier and Au–Sn bonding layers were deposited on the p-contacts and the sample was bonded to the Si substrate using a thermocompressive bonding process at 300 °C. Subsequently, the laser lift-off process of the sapphire/MQW LED/Si structure was performed in air using a Lambda Physik Compex 205 KrF pulsed excimer laser. After the LLO process, undoped GaN was removed using ICP etching. Then, the N-face GaN was photochemically etched in KOH under UV illumination.

### Implementation of subwavelength-scale nanorods

The Ag was deposited on the photochemically etched GaN using an e-beam evaporator at 6.5 kV with a 99.999% Ag granule (Taewon Scientific Co.) under 1 × 10^−6^ Torr. Afterward the Ag deposited GaN were dry etched using ICP with Cl_2_ plasma. The plasma source power was 350 W at radio frequency (RF) and chuck bias was 50 W RF and process pressure was 1 × 10^−2^ Torr. After ICP process, residual AgCl compounds were removed by dipping in HCl (OCI, 35%) 3 min. The surface morphology of the GaN was measured using a field-emission scanning electron microscope (XLS30s FEG, PHILIPS) with 5 kV accelerating voltage. To protect nanostructures, 400-nm-thick SiO_2_ was deposited using a plasma enhanced chemical vapor deposition (PECVD) with SiH_4_, N_2_O, and He. The substrate temperature was maintained with 225 °C, plasma power is 150 W, and process pressure is 200 mTorr. To form n-contact, the SiO_2_ was selectively etched using conventional photolithography and buffered oxide etcher. The 10-nm-thick Ti and 500-nm-thick Al was used as n-contact.

### Electromagnetic wave simulation

The transmittance and reflectance were calculated using a commercial electromagnetic wave calculation module (Diffractmod, Synopsys). The simulation structure consists of flat GaN substrate and GaN nanorods. The GaN nanorods were assumed to be periodic structures for efficient calculation. The gap of the nanorods was set to be half of the diameter of the nanorods. The number of harmonics for resolving nanostructures was set to be 5. Diffused transmittance was calculated by subtracting specular transmittance (normal direction) from the total transmittance. The cross-sectional electric field distributions were calculated using a finite-difference time-domain (FDTD) method with commercial module (Fullwave, Synopsys). The Gaussian plane wave was excited and the simulations were performed until steady-state. The fast Fourier transform (FFT) monitor was used to obtain spatial field distribution. To calculate far-field radiation pattern and LEE, dipole source with wavelength of 450 nm was excited in the GaN. The total width of the structure is 10 µm. The hexagonal pyramid structures had diameter of 1 µm and side wall angle of 31.6°. The geometry of the implemented nanorods was same as above. The grid size of the simulation was set to be 20 nm. The boundary condition of the calculation was set to be perfect matched layer to avoid unwanted reflection.

## Supplementary Information

Supplementary Information.
