# Supplementary material for: Subwavelength-scale nanorods implemented hexagonal pyramids structure as efficient light-extraction in Light-emitting diodes
Source: Sci Rep. 2020 Mar 26;10:5540. doi: 10.1038/s41598-020-62257-8 (PMC7098980; doi:10.1038/s41598-020-62257-8)
Supplement: Supplementary file 1 — Supplementary Information. [file 41598_2020_62257_MOESM1_ESM.docx]

**Supplementary Information**

Subwavelength-scale nanorods implemented hexagonal pyramids structure as efficient light-extraction in Light-emitting diodes

Jae Yong Park^1,†^, Buem Joon Kim^1,†^, Chul Jong Yoo^2^, Wan Jae Dong^1^, Illhwan Lee^1^, Sungjoo Kim^1^, and Jong-Lam Lee^1,2^*

[*] E-mail: jllee@postech.ac.kr

^†^ These authors contributed equally to this work

**Supplementary Table S1.** Conventional methods to increase LEE in vertical light-emitting diodes (VLEDs)

| Structure  (Method) | Enhancement factor  (vs. plarnar LED) | References |
| --- | --- | --- |
| Hexagonal pyramid (PCE) | 2.3 | ^1^ |
| Hexagonal pyramid (PCE) | 2.34 | ^2^ |
| Hexagonal pyramid (PCE) | 2.36 | ^3^ |
| Hexagonal pyramid (PCE) | 3.04 | ^4^ |
| Photonic crystal nanocone | 2.1 | ^5^ |
| ZnO nanorods | 1.39 | ^6^ |
| TiO_2_ pattern | 1.75 | ^7^ |
| N-GaN nanorods | 1.4 | ^8^ |
| ZnO nanorods | 2.5 | ^9^ |
| ZnO nanorods | 1.3 | ^10^ |
| ITO nanorods | 1.50 | ^11^ |
| High refractive index pattern | 1.28 | ^12^ |
| GaN Cone | 2.14 | ^13^ |
| GaN hole | 1.44 | ^14^ |
| Photonic crystal GaN | 1.77 | ^15^ |

1. Fujii T., Gao Y., Sharma R., Hu E., DenBaars S., Nakamura S. Increase in the extraction efficiency of GaN-based light-emitting diodes via surface roughening. *Appl. Phys. Lett.* **84**, 855-857 (2004).

2. Kim H.*, et al.* Light-extraction enhancement of vertical-injection GaN-based light-emitting diodes fabricated with highly integrated surface textures. *Opt. Lett.* **33**, 1273-1275 (2008).

3. Lee Y.-J., Kuo H.-C., Lu T.-C., Wang S.-C. High light-extraction GaN-based vertical LEDs with double diffuse surfaces. *IEEE J. Quantum Electron.* **42**, 1196-1201 (2006).

4. Son J. H., Kim J. U., Song Y. H., Kim B. J., Ryu C. J., Lee J. L. Design Rule of Nanostructures in Light‐Emitting Diodes for Complete Elimination of Total Internal Reflection. *Adv. Mater.* **24**, 2259-2262 (2012).

5. Cho H. K., Kim S.-K., Bae D. K., Kang B.-C., Lee J. S., Lee Y.-H. Laser liftoff GaN thin-film photonic crystal GaN-based light-emitting diodes. *IEEE Photonics Technol. Lett.* **20**, 2096-2098 (2008).

6. Chiu C.*, et al.* Enhancement of light output intensity by integrating ZnO nanorod arrays on GaN-based LLO vertical LEDs. *Electrochem. Solid-State Lett.* **11**, H84-H87 (2008).

7. Kim S.-K., Cho H. K., Bae D. K., Lee J. S., Park H.-G., Lee Y.-H. Efficient GaN slab vertical light-emitting diode covered with a patterned high-index layer. *Appl. Phys. Lett.* **92**, 241118 (2008).

8. Tsai M.-A.*, et al.* Efficiency enhancement and beam shaping of GaN–InGaN vertical-injection light-emitting diodes via high-aspect-ratio nanorod arrays. *IEEE Photonics Technol. Lett.* **21**, 257-259 (2009).

9. Ye B. U.*, et al.* Enhancing Light Emission of Nanostructured Vertical Light‐Emitting Diodes by Minimizing Total Internal Reflection. *Adv. Funct. Mater.* **22**, 632-639 (2012).

10. Jeong H.*, et al.* Light-extraction enhancement of a GaN-based LED covered with ZnO nanorod arrays. *Nanoscale* **6**, 4371-4378 (2014).

11. Tsai M.-A., Wang H.-W., Yu P., Kuo H.-C., Lin S.-H. High extraction efficiency of GaN-based vertical-injection light-emitting diodes using distinctive indium–tin-oxide nanorod by glancing-angle deposition. *Jpn. J. Appl. Phys.* **50**, 052102 (2011).

12. Byeon K.-J.*, et al.* Enhanced light output from vertical light-emitting diodes with an imprinted highly refractive polymer layer. *Curr. Appl Phys.* **11**, S147-S150 (2011).

13. An H.-M., Sim J. I., Shin K. S., Sung Y. M., Kim T. G. Increased light extraction from vertical GaN light-emitting diodes with ordered, cone-shaped deep-pillar nanostructures. *IEEE J. Quantum Electron.* **48**, 891-896 (2012).

14. Byeon K.-J.*, et al.* Two inch large area patterning on a vertical light-emitting diode by nano-imprinting technology. *Semicond. Sci. Technol.* **25**, 035008 (2010).

15. Huang H.-W., Lin C.-H., Huang Z.-K., Lee K.-Y., Yu C.-C., Kuo H.-C. Double photonic quasi-crystal structure effect on GaN-based vertical-injection light-emitting diodes. *Jpn. J. Appl. Phys.* **49**, 022101 (2010).


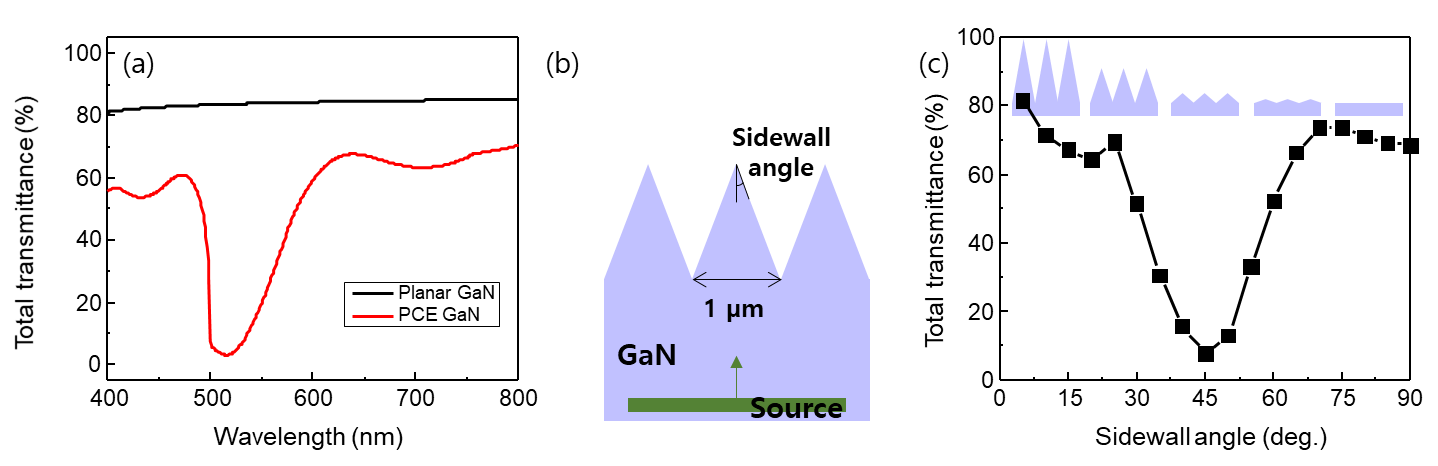


**Figure S1**. (a) Calculated total transmittance spectrum of planar GaN and photochemically etched (PCE) GaN. (b) Schematic diagram of calculated structure for various side wall angle of pyramid. (c) Calculated total transmittance as a function of side wall angle of pyramid at fixed diameter of 1 µm


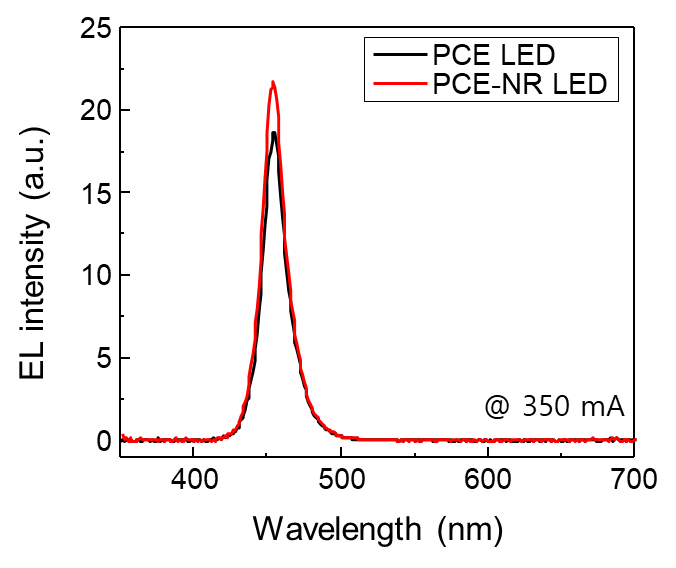


**Figure S2**. Electroluminescence (EL) spectrum with hexagonal pyramid GaN (PCE GaN), and nanorods implemented hexagonal pyramid GaN (PCE-NR GaN) at current density of 350 mA mm^-2^


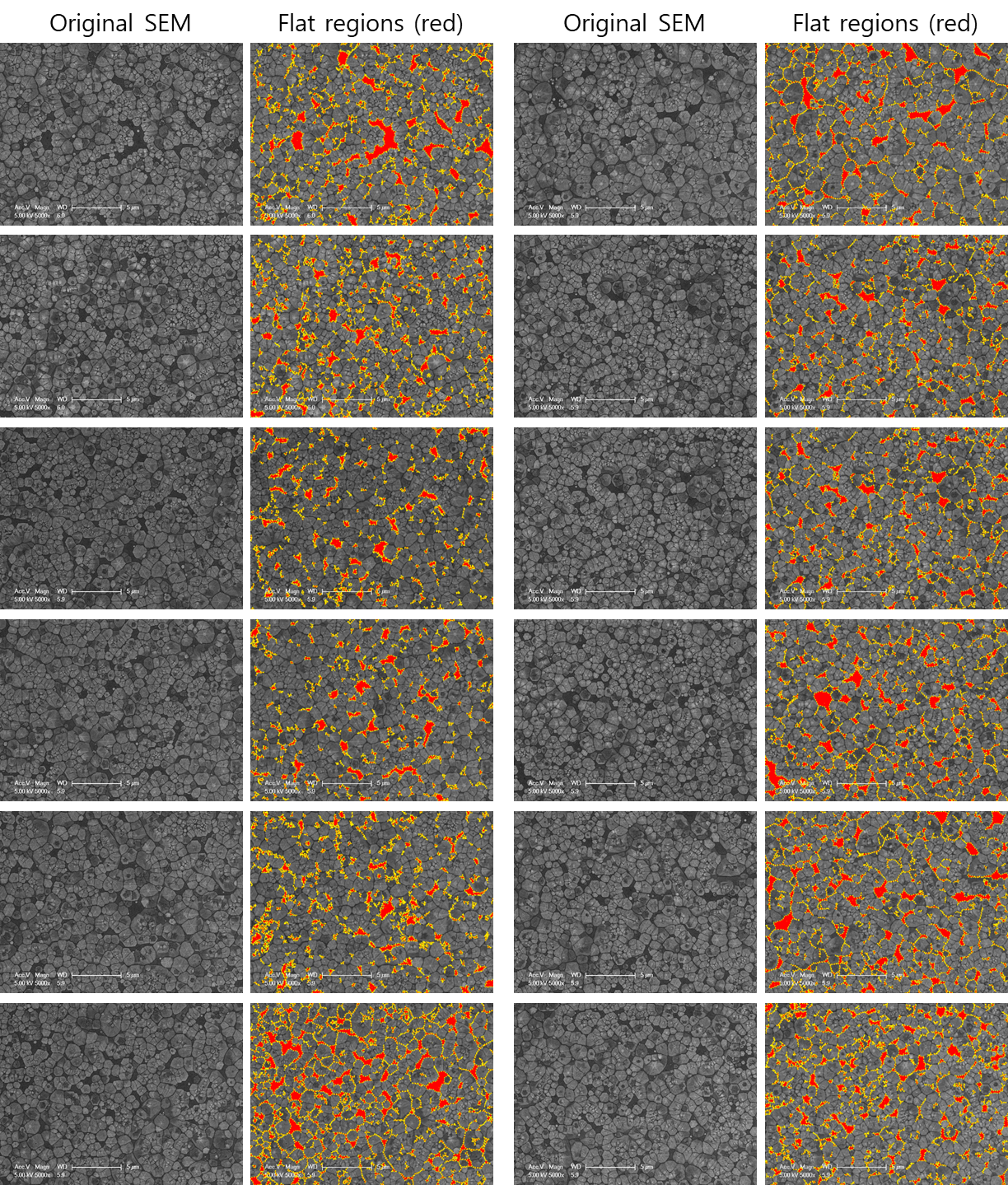


**Figure S3.** Image analysis of flat regions in photochemically etched (PCE) structures. The flat regions were filled with red color.

**
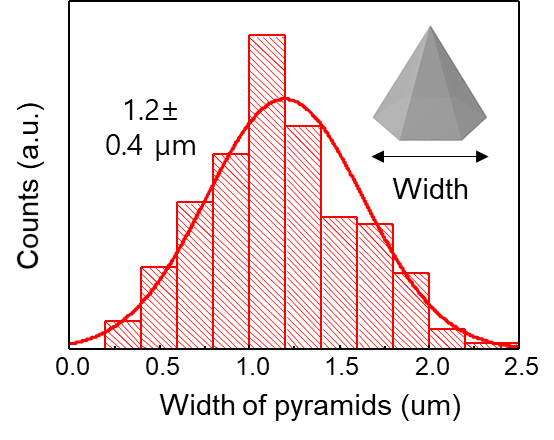
**

**Figure S4.** Histogram of width of pyramids in photochemically etched (PCE) structures.
